# Navigating the nexus of type 2 diabetes mellitus and breast cancer: a comprehensive review of co-occurrence

**DOI:** 10.3389/fonc.2025.1624896

**Published:** 2025-09-08

**Authors:** Rabiya Saroosh, Nazir Ahmad, Beenish Israr, Sana Arif, Nizwa Itrat, Abdul Momin Rizwan Ahmad

**Affiliations:** ^1^ Department of Nutrition and Dietetics, The University of Faisalabad, Faisalabad, Pakistan; ^2^ Department of Nutritional Sciences, Government College University Faisalabad, Faisalabad, Pakistan; ^3^ Institute of Home Sciences, Faculty of Food, Nutrition and Home Sciences, University of Agriculture, Faisalabad, Pakistan; ^4^ Department of Health Sciences, University of York, York, United Kingdom; ^5^ Department of Human Nutrition and Dietetics, NUST School of Health Sciences, National University of Sciences & Technology (NUST), Sector H-12, Islamabad, Pakistan

**Keywords:** epidemiological data, hyperglycemia, type 2 diabetes mellitus, breast cancer, adipokine, pro-carcinogenic environment, multidisciplinary approach

## Abstract

The co-occurrence of type 2 diabetes mellitus and breast cancer has received considerable attention due to their global prevalence and shared metabolic pathways, greatly affecting quality of life and life expectancy, especially in women. Epidemiological evidence indicates that women with type 2 diabetes mellitus have a 20-30% higher risk of developing breast cancer than women without type 2 diabetes mellitus. This review was conducted through a comprehensive and structured literature search to identify relevant peer-reviewed studies examining the relationship between type 2 diabetes mellitus and breast cancer. To ensure the quality and relevance of the included literature, only studies published in English were considered. The focus was on literature addressing pathological mechanisms, epidemiological data, and shared risk factors contributing to the coexistence of these conditions. Preference was given to recent publications, including systematic reviews, meta-analyses, and high-quality original research articles. The primary databases searched included PubMed, Scopus, Web of Science, and Google Scholar. The increased risk of breast cancer among type 2 diabetic patients is largely attributed to shared risk factors such as obesity, hyperglycemia, dietary patterns, physical inactivity, age, hormonal imbalances, and genetic predispositions, all of which contribute to the coexistence of these conditions. Chronic inflammation, hyperinsulinemia, and persistent hyperglycemia, together with dysregulation of adipokine and estrogen signaling, create a carcinogenic environment that facilitates the development of breast cancer in type 2 diabetic patients. This review emphasizes the urgent need for a multidisciplinary approach to prevention and treatment. Effective intervention strategies can reduce the dual burden of these diseases, resulting in better patient outcomes and improved quality of life.

## 1 Introduction

Non-communicable diseases (NCDs) represent the leading cause of death globally, more than all other causes. The impact of these diseases is growing faster in low-income countries than in other regions. Among NCDs, type 2 diabetes mellitus and cancer pose significant challenges to healthcare systems (1). Type 2 Diabetes mellitus is a prevalent (2), multifactorial, and heterogeneous disorder (3). It is characterized by a high insulin state resulting from insulin resistance in adipose and muscle tissues, which triggers an insufficient compensatory increase in insulin production. Over time, cellular decompensation and absolute insulin levels decrease, but this usually only occurs in the advanced stages of type 2 diabetes mellitus (4).

According to the International Diabetes Federation (IDF), type 2 diabetes mellitus is diagnosed when fasting blood glucose is ≥126 mg/dL or 2-hour plasma glucose during an oral glucose tolerance test is ≥200 mg/dL. While these criteria are essential for clinical identification, the broader concern lies in the resulting metabolic disturbances. Symptoms such as fatigue, excessive thirst, frequent urination, and numbness arise from sustained glucose imbalance. These disruptions contribute to serious long-term complications, leading to increased healthcare costs, reduced quality of life, and higher mortality rates (5).

Type 2 diabetes mellitus currently affects approximately 537 million adults aged 20 to 79 globally, representing 10.5% of all adults in this age range. By 2030, the number of people with type 2 diabetes mellitus is expected to increase to 643 million, reaching 783 million by 2045. According to the 10^th^ edition of the International Diabetes Federation (IDF), the incidence of type 2 diabetes mellitus in Southeast Asia (SEA) countries has been increasing for at least 20 years, with current estimates exceeding previous predictions (6). Worldwide, 1 in 10 and over 3 in 4 adults with type 2 diabetes mellitus live in low- and middle-income countries. In Pakistan, a developing country in South Asia, the prevalence of type 2 diabetes mellitus has reached epidemic proportions. Pakistan ranks 3rd in prevalence, affecting 30.8% of adults, 26.9% undiagnosed. Type 2 Diabetes mellitus is the 8^th^ leading cause of death worldwide, contributing to 17.5% of deaths in Pakistan (7). This trend is accompanied by increased rates of certain cancers, leading to speculation that there may be a possible direct link between type 2 diabetes mellitus and cancer. This trend is likely due to the increasing westernization of lifestyle, a trend probably shared by most Asian populations (8). The link between the two diseases was first suggested in 1934 and has been extensively researched now recognized type 2 diabetes mellitus is a risk factor for various types of cancer (9).

A substantial body of evidence now highlights a clear and consistent increase in cancer risk associated with type 2 diabetes mellitus. For type 2 diabetes mellitus, the strength of this association varies by cancer site, being stronger for pancreatic, liver, breast, bladder, endometrial, colorectal, non-Hodgkin’s lymphoma, and kidney cancers. Although the risk of stomach cancer is high in the Japanese population, this trend may not be universal. Men with type 2 diabetes mellitus typically have a 10–20% lower risk of prostate cancer, which is linked to lower circulating testosterone levels. There is limited data for other rarer cancers, hindering firm conclusions. Mortality risk is particularly high for pancreatic, colon, liver, and bladder cancers, yet data on rare cancer outcomes are scarce (10).

**Figure 1:**
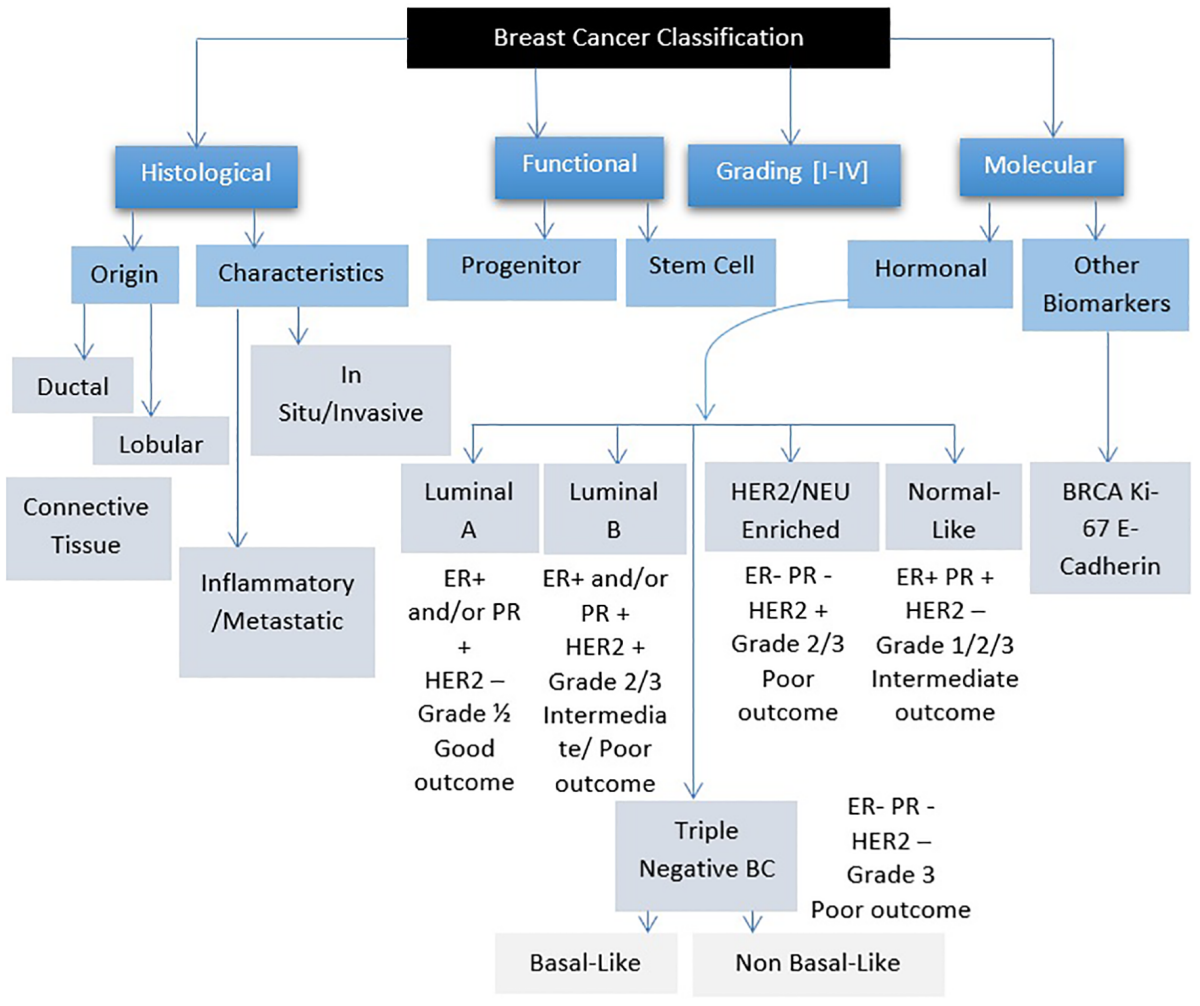
Breast cancer classification.

Breast cancer refers to a group of diseases where cells in breast tissue change and divide uncontrollably, often resulting in the formation of a lump or mass. It can be further classified based on hormone receptor profiles and other factors (**Figure 1**) (11). Most cases of breast cancer start in the milk glands (lobules) or the tubes (ducts) that connect the milk glands to the nipple. In the early stages, breast cancer usually has no symptoms, which is why breast screening plays an important role in early detection. The most common physical symptom is a painless lump, although in some cases, breast cancer can spread to the underarm lymph nodes, causing a lump or swelling to develop before the tumor becomes noticeable. Less common signs and symptoms include breast pain, heaviness, thickening, swelling, dimpling, or redness of the breast skin, changes in the nipple, sudden discharge (especially bloody), itching, or retraction. Any persistent breast changes should be evaluated by a physician (12).

Breast cancer is the most commonly diagnosed cancer in women and the fifth leading cause of cancer deaths worldwide. In 2018 alone, more than 2 million new cases were documented. Approximately 1 in 8 women (13%) and ~1 in 1000 males are diagnosed with invasive breast cancer during their lifetime, with 1 in 39 women (3%) dying of the disease. These figures represent the average risk and account for deaths from other causes that may preempt a breast cancer diagnosis. However, an individual woman’s risk is influenced by age, race or ethnicity, personal or family medical history, and reproductive history (11, 12). Pakistan ranks 1^st^ in Asia and 2^nd^ worldwide in breast cancer incidence, with 90,000 cases and 40,000 deaths reported annually. The report states that 1 out of every 9 Pakistani women is at risk of breast cancer at some point in her life (13).

Since the twentieth century, many hypotheses have proposed a link between type 2 diabetes mellitus and cancer, with increasing evidence, particularly associating type 2 diabetes mellitus with cancer risk, prognosis challenges, and treatment outcomes (14). A well-designed meta-analysis supports these findings, showing that type 2 diabetic women have a 23% higher risk of breast cancer than non-type 2 diabetic women. Similarly, another recent meta-analysis found that preexisting type 2 diabetes mellitus increases 37% all-cause mortality and a 17% increase in breast cancer mortality in women (15). The association between these diseases is due to shared risk factors (16), with various comorbidity confounders exacerbating the situation. Chronic obesity and an inactive lifestyle, which develops hyperinsulinemia, may be the underlying causes, and type 2 diabetes mellitus may be an innocent bystander (17). Additional potential confounding factors include gender, age, diet, alcohol consumption, smoking (18), and use of insulin therapy (19).

Type 2 diabetes mellitus can make the clinical course of cancer more aggressive, increase its metastatic potential, and potentially promote cancer growth by making the host less resistant to the disease. This may be due to a compromised immune system in type 2 diabetics. Furthermore, type 2 diabetes mellitus is also associated with physical frailty and reduced quality of life in cancer patients, which significantly compromises the outcome of their treatment (1).

## 2 Epidemiology links between type 2 diabetes mellitus and breast cancer

Breast cancer incidence rates are significantly higher in Western countries than in Asia, with half of all cases and 60% of associated deaths occurring in developing countries (**Table 1**). Although incidence and mortality rates have decreased in North America and parts of Europe, they continue to increase in Asian countries (27).

**Table 1:** Epidemiological data linking type 2 diabetes mellitus with breast cancer.

Study name	Country	Study type	Population/Sample size	Study period	Age group	Characteristic findings	Refs
Nurse Health Study	USA	Follow up	116,488 Nurses	1976–1988	30–55	Women with type 2 diabetes mellitus showed a slightly higher incidence of breast cancer	(20)
Long Island Breast Cancer Study Project	USA	Population-based (case-control and follow-up)	1508	1996–1997	30 +	Type 2 diabetes mellitus is associated with an increased incidence of breast cancer in older and non-white women	(21)
SEER-Medicare based study	USA	Observational Cohort	2418	2001–2007	> 80 (Mean age: 77.8)	Type 2 diabetes mellitus is associated with advanced cancers and increased mortality	(22)
Meta-analysis of type 2 diabetes mellitus and breast cancer risk	Various (North America, Europe, and Asia)	Meta-analysis (case-control and cohort)	20 Studies (30,568 cases)	1966–2007	20–95	Women with type 2 diabetes mellitus have a 20% increased risk of breast cancer	(23)
Retrospective cohort study in China	China	Retrospective Cohort	366 cases	2002–2008	mean age: 61.1	type 2 diabetes mellitus is associated with an increased risk of breast cancer	(9)
Type 2 diabetes mellitus increases the risk of breast cancer	Various	Meta-analyses (case-control and cohort)	43 studies (422,631 cases)	1990-2012	Varied	type 2 diabetes mellitus increases the risk of breast cancer in women	(24)
Meta-analysis based on a random effects model	Various (North America, Europe, and Asia)	Meta-analysis	39 studies (58,690 cases)	1993-2011	All ages	Women with type 2 diabetes mellitus have a 27% increased risk of breast (reduced to 16% after BMI adjustment)	(25)
Type 2 diabetes mellitus as a risk factor for breast cancer in women	Pakistan	Case-control study	400 patients	2014–15	30-70	17.69% of breast cancer patients reported type 2 diabetes mellitus	(26)

The link between type 2 diabetes mellitus and breast cancer was strongest in Europe (RR=1.88, 95% CI: 1.56-2.25), followed by the United States (RR=1.16, 95% CI: 1.12-1.20). In Asia, the correlation showed no significant difference (RR=1.01, 95% CI: 0.84-1.21), suggesting that ethnic and regional factors may influence breast cancer incidence. The difference in breast cancer mortality between type 2 diabetic individuals and non-type 2 diabetic individuals is greater in Asia (RR=2.05) than in the United States (RR=1.40). This disparity can be attributed to variations in access to health care, socioeconomic and educational status, lifestyle choices, and especially mammography utilization rates (28).

## 3 Potential confounding factors

### 3.1 Non-modifiable factors

#### 3.1.1 Age

Age is a non-modifiable factor that plays a crucial role in the development of both type 2 diabetes mellitus and breast cancer. As individuals age, insulin sensitivity decreases, and pancreatic beta-cell function declines, increasing the risk of type 2 diabetes mellitus. Similarly, advancing age contributes to a higher incidence of breast cancer, particularly after menopause, due to cumulative hormonal exposure and age-associated genetic mutations. Breast cancer is most common in women aged 50 years and older, largely linked to prolonged estrogen exposure and decreased immune surveillance. Recent evidence highlights that approximately 80% of breast cancer cases among individuals with type 2 diabetes mellitus occur in those aged 60 and above, whereas only 20.5% arise in the 18–59 age group. However, younger individuals with type 2 diabetes mellitus exhibit nearly double the relative risk of developing breast cancer compared to older non-type 2 diabetics (29).

#### 3.1.2 Gender

Gender significantly influences breast cancer risk in individuals with type 2 diabetes mellitus. Women with type 2 diabetes mellitus are observed to have a 20–30% higher risk of developing breast cancer compared to non-type 2 diabetic women, largely due to the interaction between hyperinsulinemia, insulin resistance, and estrogen-driven pathways. These mechanisms create a hormonal and inflammatory environment that fosters tumor growth. In contrast, breast cancer in type 2 diabetic men is rare but may be slightly more prevalent than in non-type 2 diabetic males, potentially due to obesity-induced aromatization of androgens to estrogens and altered testosterone levels. While literature on male breast cancer in type 2 diabetes mellitus remains limited, the existing data suggest the need to consider sex-specific hormonal dynamics in understanding risk patterns (30, 31).

#### 3.1.3 Height

Height has emerged as a potential risk factor for breast cancer in various populations, including type 2 diabetics. Research suggests that being tall may slightly increase breast cancer risk, regardless of type 2 diabetes mellitus status. This link is thought to be due to elevated levels of growth factors and hormones, such as insulin-like growth factor 1 (IGF-1), which play a role in cancer development. Numerous studies have established a significant correlation between tall height and breast cancer in both type 2 diabetic and non-type 2 diabetic individuals. A large analysis including more than 5 million women found that every 10-centimeter (approximately 4 inches) increase in height was associated with a 17% higher risk of breast cancer. Although the underlying mechanisms are still unclear, they may involve variations in early developmental patterns, hormonal levels, and genetic predispositions. Furthermore, height has been linked to an increased risk of several other types of cancer (32, 33).

#### 3.1.4 Genetic predisposition

Genetic predisposition is a critical determinant of breast cancer risk, especially when combined with metabolic disturbances from type 2 diabetes mellitus. Individuals with a family history of breast cancer are at significantly increased risk, and this risk may be compounded in type 2 diabetic patients. Mutations in high-penetrance genes such as BRCA1, BRCA2, TP53, PTEN, CDH1, STK11, and ATM are well-established contributors to hereditary breast cancer. Additionally, type 2 diabetes mellitus-related polymorphisms and single-nucleotide polymorphisms (SNPs) in genes regulating insulin signaling and inflammatory pathways may interact with these mutations, further elevating cancer susceptibility. The co-occurrence of type 2 diabetes mellitus and genetic mutations may enhance oxidative stress, insulin resistance, and estrogen synthesis—factors that synergistically promote carcinogenesis (34, 35).

#### 3.1.5 Race and ethnicity

Race and ethnicity play an important role in influencing breast cancer risk among individuals with type 2 diabetes mellitus, highlighting significant disparities in both incidence and outcomes between different demographic groups. Extensive research consistently indicates that African American and Hispanic women with type 2 diabetes mellitus have a 20-30% higher risk of breast cancer than their non-type 2 diabetic counterparts and other racial or ethnic groups (36). These disparities are multifaceted, including socioeconomic status, disparities in healthcare access, genetic variations, and potentially complex interactions between type 2 diabetes mellitus and breast cancer risk factors within specific racial or ethnic populations (37).

#### 3.1.6 Dense breast tissue

Dense breast tissue is a major challenge in breast cancer detection and risk assessment in type 2 diabetic patients. It is characterized by a higher proportion of fibrous and glandular tissue, which can obscure the tumors on mammograms, potentially delaying diagnoses and affecting treatment outcomes (38, 39). This issue is important for women with type 2 diabetes mellitus, as they already face an elevated risk of breast cancer due to metabolic factors associated with type 2 diabetes mellitus (40).

#### 3.1.7 Menstrual periods

The timing of reproductive milestones—such as early menarche (before age 12) and late menopause (after age 55)—plays a pivotal role in breast cancer risk, especially in women with type 2 diabetes mellitus. Extended exposure to endogenous estrogen increases the likelihood of developing hormone-receptor-positive breast cancers. In women with type 2 diabetes mellitus, metabolic dysfunctions further disrupt hormonal balance, exacerbating this risk. Premenopausal breast cancers in type 2 diabetics are often aggressive and hormone-receptor-negative, whereas postmenopausal cases are commonly hormone-receptor-positive and influenced by adipose-derived estrogen. The combination of prolonged estrogen exposure and insulin resistance creates a permissive environment for tumor development (41).

### 3.2 Modifiable factors

#### 3.2.1 Body mass index

Body Mass Index (BMI), a measure of body fat relative to height and weight, affects breast cancer risk in patients with type 2 diabetes mellitus. A high BMI level is linked to increased levels of insulin and estrogen, both of which contribute to the development of breast cancer. Research shows that every 5 kg/m² rise in Body Mass Index corresponds to a 12% higher risk of postmenopausal breast cancer (42). Women with type 2 diabetes mellitus and obesity are at higher risk, as excess weight increases metabolic disturbances and inflammation, which can lead to poorer cancer outcomes (43).

#### 3.2.2 Specific fat accumulation areas

Specific areas of fat accumulation, such as visceral fat and waist circumference, affect breast cancer risk in type 2 diabetic patients. An increase in visceral fat, which surrounds the abdominal organs, is strongly associated with insulin resistance and high inflammation levels, which contribute to increased estrogen levels and breast cancer risk (44). Studies show that central obesity, characterized by excess fat around the abdomen, is particularly harmful in people with type 2 diabetes mellitus, as it exacerbates metabolic disorders and hormonal imbalances (45). Research has highlighted that every 5 cm increase in waist circumference is associated with a 7% higher breast cancer risk in postmenopausal women (46).

#### 3.2.3 Dietary habits

Recent studies indicate an important role of dietary habits in influencing the risk of breast cancer in type 2 diabetic individuals. A cohort study involving 10,000 participants over ten years indicated that a higher intake of fish, eggs, leafy vegetables, and nuts was linked to a lower breast cancer risk (47). These nutritious foods, which are abundant in omega-3 fatty acids, antioxidants, and essential vitamins, provide a protective effect against breast cancer in type 2 diabetic individuals. Additionally, a meta-analysis supports these findings, suggesting that a diet emphasizing these food groups may significantly reduce breast cancer risk in type 2 diabetic women (48).

A population-based cohort study of 20,000 women aged 40–70 years, followed for 15 years, found an association between dietary patterns and risk of breast cancer. Participants completed a detailed dietary assessment using a food frequency questionnaire. During the study period, 1,500 cases of breast cancer were diagnosed. Analysis revealed that higher intakes of red meats (HR = 1.18, 95% CI 1.04-1.34), processed meats (HR = 1.25, 95% CI 1.10-1.42), and sugary drinks (HR = 1.32, 95% CI 1.16-1.50) were associated with elevated risk of breast cancer. In contrast, a diet rich in whole grains, vegetables, fruits, and lean protein was associated with a lower risk (HR = 0.85, 95% CI 0.76-0.95). These findings highlight the importance of dietary changes in lowering breast cancer risk within the studied population (49).

In a case-control study conducted in northern Alberta, Canada, researchers investigated the relationship between dietary factors and breast cancer risk in 577 women diagnosed with breast cancer in 1976-77, alongside 826 age-stratified female controls without the disease. Participants were queried about specific aspects of their diet. This study found significant trends in relative risks (RRs) across tertiles of consumption for several nutrients. Increasing frequency of beef consumption showed RRs of 1.0, 2.3, and 1.5 (test for trend, p < 0.001), indicating a significant trend towards increased risk with higher intake. Similarly, pork consumption demonstrated RRs of 1.0, 1.6, and 2.2 (test for trend, p < 0.001), indicating a significant link with breast cancer risk. Consumption of sweet desserts also showed a trend with RRs of 1.0, 1.3, and 1.5 (test for trend, p = 0.01), suggesting increased risk associated with higher intake levels. Additionally, greater risks were observed in using table butter and frying with butter or margarine than with vegetable oils (50).

#### 3.2.4 Aluminum foil and utensils

Studies have suggested mechanisms by which aluminum exposure may contribute to cancer development. Aluminum salts, commonly consumed through cooked food or stored in aluminum foil, have estrogen-like effects *in vitro*, possibly affecting hormone-sensitive cancers such as breast cancer (51). Additionally, while aluminum foil is generally not used directly for cooking acidic or salty foods because of its reactive properties, evidence suggests that aluminum can leach into food, especially when heated (52). In contrast, comprehensive reviews and epidemiological studies have found no significant association between exposure to aluminum from foil or utensils and breast cancer risk. A systematic review concluded that current evidence does not establish a direct causal relationship between aluminum exposure from cookware or other sources and breast cancer (53).

#### 3.2.5 Repeated use of same oil for cooking

Repeated use of the same oil, especially when exposed to high temperatures and repeated heating, leads to the formation of harmful substances like polycyclic aromatic hydrocarbons (PAHs) and advanced glycation end products (AGEs). PAHs are produced during the incomplete combustion of organic matter, including oil when heated to high temperatures. AGEs, on the other hand, are compounds formed by the reaction of sugars with proteins, lipids, or nucleic acids during the cooking processes at high temperatures. Both PAHs and AGEs have been implicated in oxidative stress, inflammation, and cancer development. For individuals with type 2 diabetes mellitus, who already face an elevated risk of breast cancer due to metabolic and hormonal factors, additional exposure to these carcinogenic compounds from reused oil may further increase their risk. Therefore, adopting cooking practices that include oil rotation, moderate heating, and choosing oils with high smoke points and healthy fatty acid profiles can help reduce these risks (54).

#### 3.2.6 Physical activity

Research shows that a sedentary lifestyle contributes to insulin resistance and high levels of circulating insulin and insulin-like growth factors, both of which correlate with an increased breast cancer risk. Type 2 Diabetics with low physical activity levels often struggle to maintain a healthy weight, increasing their breast cancer risk. For instance, a meta-analysis found a positive association between low physical activity levels and breast cancer risk in type 2 diabetic individuals. Therefore, promoting regular physical activity in type 2 diabetic individuals is important not only for type 2 diabetes management but also for reducing breast cancer risk, highlighting the importance of lifestyle modification as a key component in health management strategies (25, 55).

#### 3.2.7 Stress

Chronic stress triggers a cascade of hormonal and immune responses that can contribute to cancer progression. Chronic stress interferes with the hypothalamic-pituitary-adrenal (HPA) axis, resulting in increased production of stress hormones, particularly cortisol. These hormones, in turn, disrupt immune function and promote chronic inflammation, creating a favorable environment for tumor growth and metastasis. Psychologically, chronic stress can also contribute to unhealthy behaviors including poor dietary choices, alcohol consumption and, smoking, which are linked to an increased cancer risk. Moreover, stress can increase insulin resistance and glucose dysregulation, which are common features of type 2 diabetes mellitus, thereby increasing the breast cancer risk in type 2 diabetics (56, 57).

#### 3.2.8 Disrupted sleep cycle

A recent meta-analysis reported that short-term night shift work was associated with a modest increase in the risk of breast cancer (58), with the highest risk found in individuals who worked shifts during early adulthood (59, 60). Exposure to light at night disrupts the production of melatonin, a hormone that regulates sleep. Experimental evidence suggests that melatonin can inhibit the growth of small, established tumors and prevent new tumors (61). A 2019 review of human and animal studies by the International Agency for Research on Cancer concluded that “night shift work,” as distinct from “shift work” (identified in 2007), is carcinogenic to humans due to its association with cancers such as breast, prostate, and colorectal (62).

#### 3.2.9 Metabolic syndrome

Metabolic syndrome, a set of biological abnormalities including obesity, dyslipidemia, hypertension, and insulin resistance, often leading to type 2 diabetes mellitus, has been recognized as a contributing factor to Triple-negative breast cancer (TNBC). This association was highlighted in a case-control study of 555 West African women. Additionally, a retrospective study of 1416 type 2 diabetic breast cancer patients diagnosed between 2015 and 2020 showed a strong association between poor blood sugar management and an elevated risk of TNBC. Supporting this, an Indian clinical study found that TNBC patients who underwent seven cycles of neoadjuvant chemotherapy exhibited higher biomarkers of metabolic syndrome, including type 2 diabetes mellitus, than untreated patients (44).

#### 3.2.10 Alcohol

About 16% of breast cancer cases in the United States are linked to alcohol (63). The risk of breast cancer in women increases by about 7%-10% for every 10 grams of alcohol consumed per day (about one drink). Women who drink 2 to 3 alcoholic drinks per day have a 20% greater breast cancer risk than women who do not drink alcohol (64). Although the exact mechanism is not fully understood, alcohol may increase risk by raising levels of estrogen and other hormone, or by increasing the density of breast tissue (65).

#### 3.2.11 Tobacco

Growing evidence suggests that smoking may slightly increase the risk of breast cancer, particularly in women who have smoked for many years or started smoking at a young age (66). A family history of breast cancer can further increase this risk (67). Additionally, research shows that exposure to secondhand smoke, especially during childhood, may also contribute to the risk of breast cancer in the future (68).

#### 3.2.12 Environmental chemicals and pollutants

Several occupational, environmental, and chemical exposures have been suggested as potential causes of breast cancer. However, epidemiological studies have generally found no clear association between environmental pollutants and breast cancer risk. Research has shown no link between high levels of organochlorines, such as DDT, in the blood or fat tissue of adults and breast cancer risk (69). However, exposure to DDT during critical stages of development, such as *in utero*, during infancy, or before puberty, has been linked to an increased breast cancer risk later in life (70). Animal studies show that long-term, high-dose exposure to certain chemicals can promote the development of mammary tumor, but it is not yet clear whether low levels of exposure in the general environment do the same. Many of these chemicals have not been well studied in humans, making this an active area of research (71).

#### 3.2.13 Endocrine disruptors

Endocrine disruptors, often consisting of compounds such as phthalates, parabens, and triclosan, are known to mimic or interfere with hormones like androgen and estrogen, disrupting normal hormonal signaling pathways. Phthalates are a group of chemicals found in plastics, fragrances, and personal care products that have been linked to hormone disruption, specifically affecting estrogen and testosterone levels. They have been linked to reproductive and developmental problems and are being investigated for potential health effects (72). Parabens are widely used as preservatives in cosmetics and personal care products to inhibit microbial growth. They have been detected in human breast tissue samples and are suspected of having a role in breast cancer development due to their estrogenic properties and ability to penetrate the skin (73). Triclosan, an antimicrobial agent used in some deodorants and toothpaste, has also raised concerns about its role in endocrine disruptors and antibiotic resistance (74). Formaldehyde-releasing preservatives in hair dyes and other products release carcinogenic formaldehyde, while aromatic amines in hair dyes are classified as possible human carcinogens (75).

#### 3.2.14 Breastfeeding

Most research shows that breastfeeding for a year or more slightly decreases the breast cancer risk in women, with greater reductions seen with longer durations. A review of 47 studies from 30 countries found that the risk of breast cancer decreases by 4% for every 12 months of breastfeeding. It works by suppressing ovulation, thereby reducing lifelong exposure to estrogen, the hormone associated with breast cancer development. It promotes breast tissue maturation, aids in postpartum weight loss, and improves insulin sensitivity (76).

#### 3.2.15 Polycystic ovary syndrome

Polycystic ovary syndrome (PCOS) is marked by hormonal imbalances, such as increased androgen levels and insulin resistance, which can lead to breast cancer risk. Insulin resistance, a common feature of PCOS, leads to high circulating insulin levels and increased production of insulin-like growth factor (IGF), both of which are associated with the development and progression of breast cancer. Additionally, the chronic inflammation and hormonal dysregulation seen in PCOS may create a favorable environment for cancer cell growth (77).

#### 3.2.16 Oral contraceptives

Most research shows that current or recent use of oral contraceptives (combined estrogen and progesterone) is associated with a relatively small (about 20%) increased risk of breast cancer, especially among women who have had it before their first pregnancy. Studies of progestin-only intrauterine devices have had mixed results, but a large Denmark study linked its use to a 20% increased risk of breast cancer. On the other hand, the injectable progestin-only contraceptive, depot-medroxyprogesterone acetate (Depo-Provera), has not been linked to breast cancer, although the sample size may have been too small to detect a clear association (78). The risk is reduced when women stop using for at least 10 years, as are never users. Data on “ultra-low-dose” (20 micrograms) estrogen formulations are limited and less clear (79). Overall, it is estimated that one additional case of breast cancer is diagnosed for every 7,690 women who use hormonal contraception for one year (78).

## 4 Pathophysiology

**Figure 2:**
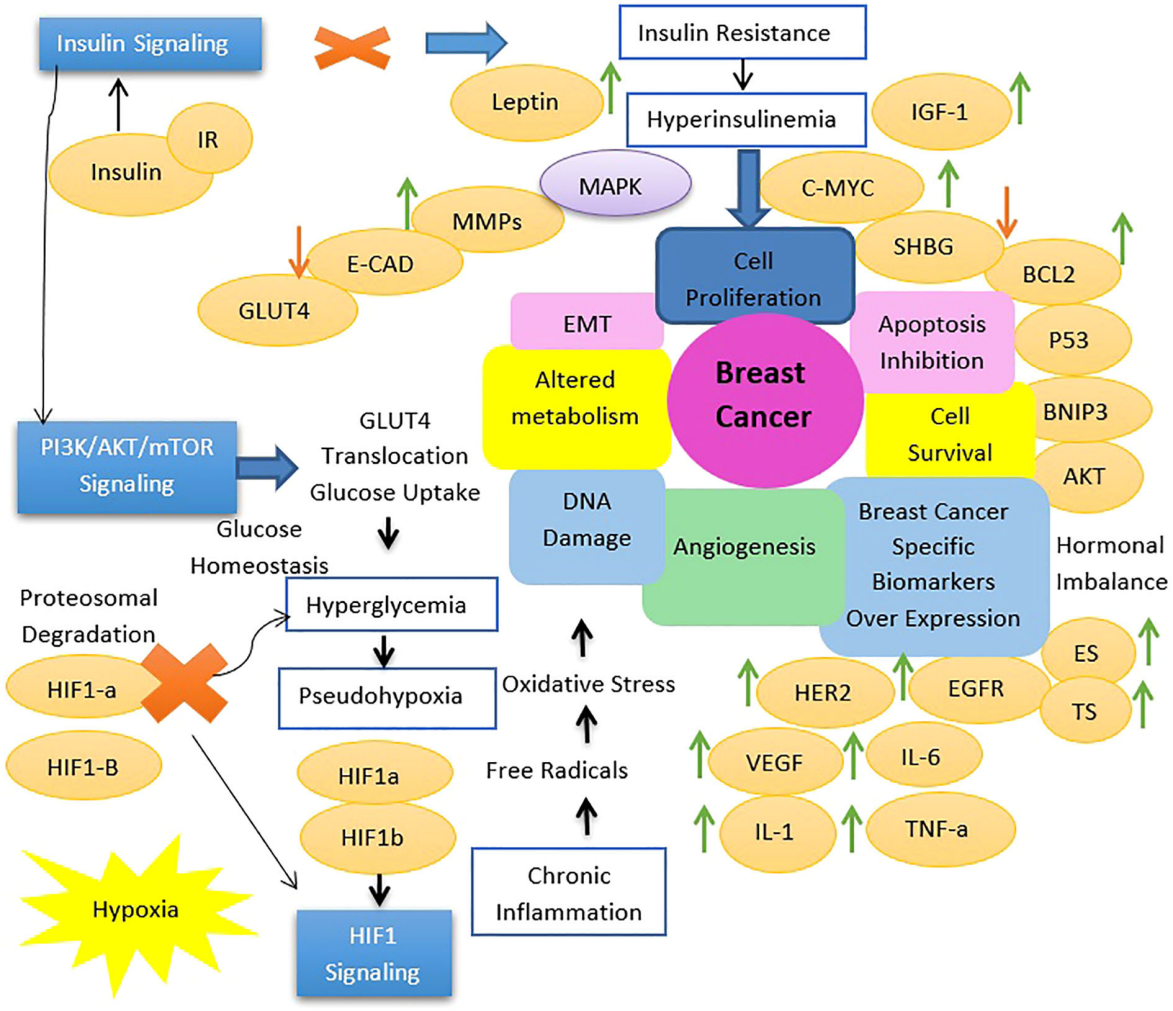
Transition in cell signaling associated with type 2 diabetes mellitus induced breast tumorigenesis.

Insulin signaling is important for maintaining metabolic homeostasis by finely regulating glucose uptake, glycogen synthesis, and lipid metabolism in response to varying blood glucose levels. When blood glucose rises, insulin binds its receptor on target cells, initiating autophosphorylation and subsequent recruitment of insulin receptor substrates (IRS) and Shc proteins. IRS activates the PI3K-AKT pathway, which is important for glucose transport, glycogen synthesis, and lipid metabolism, ensuring efficient utilization or storage of glucose and suppression of lipolysis, autophagy, proteasomal activity, and apoptosis under adequate insulin levels. In addition, Shc proteins activate the MAPK pathway, regulating cellular proliferation, differentiation, and gene transcription. Disruption of insulin signaling, as seen in insulin resistance and type 2 diabetes mellitus, leads to impaired glucose uptake and dysregulated lipid metabolism, which contributes to metabolic dysfunction and hyperglycemia. Beyond metabolic regulation, insulin and insulin-like growth factors affect cancer biology by activating the HIF1 signaling pathway. This pathway, critical for cellular adaptation to hypoxic conditions in solid tumors, regulates glycolysis, angiogenesis, and cell survival. Insulin-mediated activation of HIF1 signaling highlights its role in promoting cancer cell survival, proliferation, and adaptation to the anti-tumor microenvironments, highlighting the complex interplay between metabolic processes and cancer progression (**Figure 2**) (11) In the case of insulin resistance, it is important to note that different types of insulin-activated, signaling pathways identify an important critical distinction. The primary metabolic effects of insulin are mediated through the PI3K-Akt pathway; however, it is in this pathway that insulin resistance presents problems.

As for the MAPK signaling pathway, it would be quite intact in such a situation. Quite likely, this demonstration of signaling still being conferred by insulin would indicate that there was a shift toward this pathway to preferentially activate it. Because the MAPK pathway is largely concerned with growth, proliferation, and differentiation of the cell, rather than any of the metabolic functions associated with insulin, this would concern what is endorsing the preferential activation of this pathway in insulin resistance (35, 80, 81). Thus, the most preferred activation of the MAPK pathway for insulin resistance has real physiological consequences at that level. This then means that, if elevated, MAPK signaling could be contributing a lot to increased cell proliferation and survival, thereby contributing to tumor growth and progression, and this phenomenon can be observed in a few insulin-resistant states (80, 82, 83).

Some key breast cancer genes, like BRCA1/2, TP53, PTEN, CDH1, and STK11, significantly correlate with Type 2 Diabetes (type 2 diabetes mellitus. BRCA1/2 mutations raise type 2 diabetes mellitus risk, with high BMI being one potential factor via metabolic misregulation. TP53, a tumor suppressor, also controls metabolism, and its high level in type 2 diabetes mellitus patients suggests a common stress response. PTEN acts directly on insulin signaling active in both breast cancer and type 2 diabetes mellitus via the PI3K/Akt pathway. Likewise, some early connections of CDH1 (cell adhesion) and STK11 (metabolic LKB1 regulator) with type 2 diabetes mellitus establish common pathways for both diseases. This profound genetic interplay suggests possible common molecular mechanisms between breast cancer and type 2 diabetes mellitus. Thus, such an integrated health management approach should be warranted (84–86).

## 5 Barriers to breast cancer diagnosis in people with type 2 diabetes mellitus

Major barriers to early diagnosis and treatment of breast cancer among rural Pakistani women are limited awareness, geographical difficulties, and financial limitations. A widespread lack of awareness of breast cancer symptoms and screening methods is associated with delays in diagnosis and treatment. This lack of knowledge is exacerbated by the geographical isolation of rural areas, where limited infrastructure and transportation difficulties prevent timely access to healthcare services. Women often have to travel long distances for diagnostic and treatment facilities, such as mammography and chemotherapy. Additionally, financial barriers pose considerable challenges, as diagnostic tests and treatments are often prohibitively expensive for economically disadvantaged families, forcing some women to forgo essential medical care or even sell assets to cover expenses, thereby perpetuating poverty. Furthermore, socio-cultural norms and economic dependence limit women’s decision-making power regarding their health. Addressing these barriers requires comprehensive strategies, including better health education, expanding healthcare infrastructure in rural areas, and providing financial support to ensure equitable access to breast cancer screening and treatment. Efforts should focus on empowering women with knowledge, improving accessibility, and reducing the financial burdens for early diagnosis and better outcomes of breast cancer in rural populations in Pakistan (87, 88).

## 6 Strategies to overcome type 2 diabetes mellitus and breast cancer

Overcoming breast cancer in type 2 diabetic patients requires tailored strategies that address the specific needs of the situation, taking into account the disparities between developed and developing countries (89). There is an urgent need to reduce modifiable risk factors and increase screening through comprehensive national policies, community initiatives, and individual behavioral interventions. Although this strategy is effective but underutilized, especially in low socioeconomic areas. Tobacco taxes have significantly reduced smoking among people of low socioeconomic status, but taxes in the U.S. are still below WHO recommendations. Improving access to healthy and affordable food in areas of low socioeconomic status is essential to address healthcare disparities. Barriers to health care services, targeted communication efforts are needed to reduce cultural and language barriers. Cancer prevention efforts focus on reducing tobacco use and obesity and increasing screening and vaccination. A systematic approach that includes low socioeconomic status and racial/ethnic minorities is essential, with greater emphasis on vulnerable groups (90, 91).

Additionally, ongoing efforts in cancer epidemiology, including occupational exposures, environmental factors, and genetics, are important components of public health programs to promote healthy interventions. However, more research is needed to understand changes in cancer-related attitudes and their impact on cancer incidence and survival rates. Recognizing the challenges of behavior change, policymakers and public health professionals should consider interventions at multiple levels, including the individual level, community, and systemic perspectives (92).

## 7 Conclusion

The interplay between type 2 diabetes mellitus and breast cancer highlights the urgent need for targeted interventions. Combined risk factors such as obesity, hyperglycemia, and hormonal imbalances create a cancer-prone environment in type 2 diabetic women. Addressing chronic inflammation and metabolic dysregulation is key to disrupting this connection. A multidisciplinary approach, combining lifestyle changes, early screening, and personalized treatment, holds promise for reducing the double burden of these diseases. Focusing on prevention and comprehensive care will not only save lives but also improve the quality of life for those affected worldwide.

## References

[1] Wolde HF Molla MD Aragie H Adugna DG Teferi ET Melese EB. High burden of diabetes and prediabetes among cancer patients at University of Gondar comprehensive specialized hospital, Northwest Ethiopia. Sci Rep. (2023) 13:9431. doi: 10.1038/s41598-023-36472-y, PMID: 37296304 PMC10256839

[2] Tomic D Shaw JE Magliano DJ. The burden and risks of emerging complications of diabetes mellitus. Nat Rev Endocrinol. (2022) 18:525–39. doi: 10.1038/s41574-022-00690-7, PMID: 35668219 PMC9169030

[3] Cignarelli A Genchi VA Caruso I Natalicchio A Perrini S Laviola L. Diabetes and cancer: Pathophysiological fundamentals of a ‘dangerous affair’. Diabetes Res Clin Practice. (2018) :143:378–88. doi: 10.1016/j.diabres.2018.04.002, PMID: 29679627

[4] Wolf I Sadetzki S Catane R Karasik A Kaufman B. Diabetes mellitus and breast cancer. Lancet Oncol. (2005) 6:103–11. doi: 10.1016/S1470-2045(05)01736-5, PMID: 15683819

[5] Eketunde AO. Diabetes as a risk factor for breast cancer. Cureus. (2020) 12. doi: 10.7759/cureus.8010, PMID: 32528752 PMC7279688

[6] Kumar A Gangwar R Ahmad Zargar A Kumar R Sharma A. Prevalence of diabetes in India: A review of IDF diabetes atlas 10th edition. Curr Diabetes Rev. (2024) 20:105–14. doi: 10.2174/1573399819666230413094200, PMID: 37069712

[7] International Diabetes Federation. IDF Diabetes Atlas (2021). Available online at: https://diabetesatlas.org/idfawp/resource-files/2021/07/IDF_Atlas_10th_Edition_2021.pdf (Accessed July 17, 2025).

[8] Noto H Goto A Tsujimoto T Osame K Noda M. Latest insights into the risk of cancer in diabetes. J Diabetes Invest. (2013) 4:225–32. doi: 10.1111/jdi.12068, PMID: 24843658 PMC4015656

[9] Zhang PH Chen ZW Lv D Xu YY Gu WL Zhang XH. Increased risk of cancer in patients with type 2 diabetes mellitus: a retrospective cohort study in China. BMC Public Health. (2012) 12:1–6. doi: 10.1186/1471-2458-12-567, PMID: 22839452 PMC3487805

[10] Harding JL Shaw JE Peeters A Cartensen B Magliano DJ. Cancer risk among people with type 1 and type 2 diabetes: disentangling true associations, detection bias, and reverse causation. Diabetes Care. (2015) 38:264–70. doi: 10.2337/dc14-1996, PMID: 25488912

[11] Durrani IA Bhatti A John P. The prognostic outcome of ‘type 2 diabetes mellitus and breast cancer’association pivots on hypoxia-hyperglycemia axis. Cancer Cell Int. (2021) 21:351. doi: 10.1186/s12935-021-02040-5, PMID: 34225729 PMC8259382

[12] American Cancer Society. Breast Cancer Facts & Figures 2022-2024. Atlanta: American Cancer Society, Inc (2022). Available online at: https://www.cancer.org/content/dam/cancer-org/research/cancer-facts-and-statistics/breast-cancer-facts-and-figures/2022-2024-breast-cancer-fact-figures-acs.pdf (Accessed July 17, 2025).

[13] Munawwar A Sajjad A Faisal S Rasul A Zarbab A Bibi A. Basic findings of incidence of breast cancer in allied hospital faisalabad, Pakistan: A retrospective study. Iranian J Public Health. (2023) 52:1199. doi: 10.18502/ijph.v52i6.13000, PMID: 37484147 PMC10362829

[14] Ben Q Xu M Ning X Liu J Hong S Huang W. Diabetes mellitus and risk of pancreatic cancer: a meta-analysis of cohort studies. Eur J Cancer. (2011) 47:1928–37. doi: 10.1016/j.ejca.2011.03.003, PMID: 21458985

[15] Zhao XB Ren GS. Diabetes mellitus and prognosis in women with breast cancer: a systematic review and meta-analysis. Medicine. (2016) 95:e5602. doi: 10.1097/MD.0000000000005602, PMID: 27930583 PMC5266055

[16] Pearson-Stuttard J Zhou B Kontis V Bentham J Gunter MJ Ezzati M. Worldwide burden of cancer attributable to diabetes and high body-mass index: a comparative risk assessment. Lancet Diabetes Endocrinol. (2018) 6:e6–15. doi: 10.1016/S2213-8587(17)30366-2, PMID: 29803268 PMC5982644

[17] Renehan AG Tyson M Egger M Heller RF Zwahlen M. Body-mass index and incidence of cancer: a systematic review and meta-analysis of prospective observational studies. Lancet. (2008) 371:569–78. doi: 10.1016/S0140-6736(08)60269-X, PMID: 18280327

[18] Noto H Raskin P. Hepatitis C infection and diabetes. J Diabetes Its Complications. (2006) 20:113–20. doi: 10.1016/j.jdiacomp.2006.01.001, PMID: 16504840

[19] McCall JL Tuckey JA Parry BR. Serum tumour necrosis factor alpha and insulin resistance in gastrointestinal cancer. Br J Surg. (1992) 79:1361–3. doi: 10.1002/bjs.1800791240, PMID: 1486441

[20] Michels KB Solomon CG Hu FB Rosner BA Hankinson SE Colditz GA. Type 2 diabetes and subsequent incidence of breast cancer in the Nurses’ Health Study. Diabetes Care. (2003) 26:1752–8. doi: 10.2337/diacare.26.6.1752, PMID: 12766105

[21] Cleveland RJ North KE Stevens J Teitelbaum SL Neugut AI Gammon MD. The association of diabetes with breast cancer incidence and mortality in the Long Island Breast Cancer Study Project. Cancer Causes Control. (2012) 23:1193–203. doi: 10.1007/s10552-012-9989-7, PMID: 22674293 PMC3383781

[22] Griffiths RI Danese MD Gleeson ML Valderas JM. Epidemiology and outcomes of previously undiagnosed diabetes in older women with breast cancer: an observational cohort study based on SEER-Medicare. BMC Cancer. (2012) 12:1–4. doi: 10.1186/1471-2407-12-613, PMID: 23259613 PMC3575284

[23] García-Jiménez C Gutiérrez-Salmerón M Chocarro-Calvo A García-Martinez JM Castaño A de la Vieja A. From obesity to diabetes and cancer: epidemiological links and role of therapies. Br J Cancer. (2016) 114:716–22. doi: 10.1038/bjc.2016.37, PMID: 26908326 PMC4984860

[24] Hardefeldt PJ Edirimanne S Eslick GD. Diabetes increases the risk of breast cancer: a meta-analysis. Endocrine Related Cancer. (2012) 19:793. doi: 10.1530/ERC-12-0242, PMID: 23035011

[25] Boyle P Boniol M Koechlin A Robertson C Valentini F Coppens K. Diabetes and breast cancer risk: a meta-analysis. Br J Cancer. (2012) 107:1608–17. doi: 10.1038/bjc.2012.414, PMID: 22996614 PMC3493760

[26] Tabassum I Mahmood H Faheem M. Type 2 diabetes mellitus as a risk factor for female breast cancer in the population of Northern Pakistan. Asian Pacific J Cancer Prev. (2016) 17:3255–8. doi: 10.14456/apjcp.2016.84/APJCP.2016.17.7.3255, PMID: 27509959

[27] Zhou Y Zhang X Gu C Xia J. Influence of diabetes mellitus on mortality in breast cancer patients. ANZ J Surg. (2015) 85:972–8. doi: 10.1111/ans.12877, PMID: 25312511

[28] Liao S Li J Wei W Wang L Zhang Y Li J. Association between diabetes mellitus and breast cancer risk: a meta-analysis of the literature. Asian Pac J Cancer Prev. (2011) 12:1061–5., PMID: 21790252

[29] Ferlay J Soerjomataram I Dikshit R Eser S Mathers C Rebelo M. Cancer incidence and mortality worldwide: sources, methods and major patterns in GLOBOCAN 2012. Int J Cancer. (2015) 136:E359–86. doi: 10.1002/ijc.29210, PMID: 25220842

[30] Lipscombe LL Goodwin PJ Zinman B McLaughlin JR Hux JE. Diabetes mellitus and breast cancer: a retrospective population-based cohort study. Breast Cancer Res Treat. (2006) 98:349–56. doi: 10.1007/s10549-006-9172-5, PMID: 16541321

[31] Smith U Gale EA. Does diabetes therapy influence the risk of cancer? Diabetologia. (2009) :52:1699–708. doi: 10.1007/s00125-009-1441-5, PMID: 19597799

[32] Amadou A Ferrari P Muwonge R Moskal A Biessy C Romieu I. Overweight, obesity and risk of premenopausal breast cancer according to ethnicity: a systematic review and dose-response meta-analysis. Obes Rev. (2013) 14:665–78. doi: 10.1111/obr.12028, PMID: 23615120

[33] Kabat GC Heo M Kamensky V Miller AB Rohan TE. Adult height in relation to risk of cancer in a cohort of Canadian women. Int J Cancer. (2013) 132:1125–32. doi: 10.1002/ijc.27704, PMID: 22753236

[34] Antoniou A Pharoah PD Narod S Risch HA Eyfjord JE Hopper JL. Average risks of breast and ovarian cancer associated with BRCA1 or BRCA2 mutations detected in case series unselected for family history: a combined analysis of 22 studies. Am J Hum Genet. (2003) 72:1117–30. doi: 10.1086/375033, PMID: 12677558 PMC1180265

[35] Zhang Y Ding Y Zhu N Mi M Lu Y Zheng J. Emerging patterns and trends in global cancer burden attributable to metabolic factors, based on the Global Burden of Disease Study 2019. Front Oncol. (2023) 13:1032749. doi: 10.3389/fonc.2023.1032749, PMID: 36741020 PMC9893408

[36] Newman LA Kaljee LM. Health disparities and triple-negative breast cancer in African American women: a review. JAMA Surg. (2017) 152:485–93. doi: 10.1001/jamasurg.2017.0005, PMID: 28355428

[37] Jones BA Kasl SV Howe CL Lachman M Dubrow R Curnen MM. African-American/White differences in breast carcinoma: p53 alterations and other tumor characteristics. Cancer: Interdiscip Int J Am Cancer Society. (2004) 101:1293–301. doi: 10.1002/cncr.20500, PMID: 15368321

[38] Boyd NF Guo H Martin LJ Sun L Stone J Fishell E. Mammographic density and the risk and detection of breast cancer. New Engl J Med. (2007) 356:227–36. doi: 10.1056/NEJMoa062790, PMID: 17229950

[39] Sprague BL Gangnon RE Burt V Trentham-Dietz A Hampton JM Wellman RD. Prevalence of mammographically dense breasts in the United States. J Natl Cancer Inst. (2014) 106:dju255. doi: 10.1093/jnci/dju255, PMID: 25217577 PMC4200066

[40] Ma H Wang Y Sullivan-Halley J Weiss L Burkman RT Simon MS. Breast cancer receptor status: do results from a centralized pathology laboratory agree with SEER registry reports? Cancer Epidemiol Biomarkers Prev. (2009) 18:2214–20. doi: 10.1158/1055-9965.EPI-09-0301, PMID: 19661080 PMC3782852

[41] Colditz GA Bohlke K Berkey CS. Breast cancer risk accumulation starts early: prevention must also. Breast Cancer Res Treat. (2014) 145:567–79. doi: 10.1007/s10549-014-2993-8, PMID: 24820413 PMC4079839

[42] Chan DS Vieira AR Aune D Bandera EV Greenwood DC McTiernan A. Body mass index and survival in women with breast cancer—systematic literature review and meta-analysis of 82 follow-up studies. Ann Oncol. (2014) 25:1901–14. doi: 10.1093/annonc/mdu042, PMID: 24769692 PMC4176449

[43] Arnold M Pandeya N Byrnes G Renehan AG Stevens GA Ezzati M. Global burden of cancer attributable to high body-mass index in 2012: a population-based study. Lancet Oncol. (2015) 16:36–46. doi: 10.1016/S1470-2045(14)71123-4, PMID: 25467404 PMC4314462

[44] Harvie M Hooper L Howell AH. Central obesity and breast cancer risk: a systematic review. Obes Rev. (2003) 4:157–73. doi: 10.1046/j.1467-789X.2003.00108.x, PMID: 12916817

[45] Iyengar NM Hudis CA Dannenberg AJ. Obesity and cancer: local and systemic mechanisms. Annu Rev Med. (2015) 66:297–309. doi: 10.1146/annurev-med-050913-022228, PMID: 25587653

[46] Pischon T Lahmann PH Boeing H Tjønneland A Halkjær J Overvad K. Body size and risk of renal cell carcinoma in the European Prospective Investigation into Cancer and Nutrition (EPIC). Int J Cancer. (2006) 118:728–38. doi: 10.1002/ijc.21398, PMID: 16094628

[47] Zhang CX Ho SC Fu JH Cheng SZ Chen YM Lin FY. Dietary patterns and breast cancer risk among Chinese women. Cancer Causes Control. (2011) 22:115–24. doi: 10.1007/s10552-010-9681-8, PMID: 21080051

[48] Farvid MS Chen WY Michels KB Cho E Willett WC Eliassen AH. Fruit and vegetable consumption in adolescence and early adulthood and risk of breast cancer: population based cohort study. BMJ. (2016) 353:1–12. doi: 10.1136/bmj.i2343, PMID: 27170029 PMC5068921

[49] Bessaoud F Daures JP Gerber M. Dietary factors and breast cancer risk: a case control study among a population in Southern France. Nutr Cancer. (2008) 60:177–87. doi: 10.1080/01635580701649651, PMID: 18444149

[50] Lubin JH Burns PE Blot WJ Ziegler RG Lees AW Fraumeni JF Jr. Dietary factors and breast cancer risk. Int J Cancer. (1981) 28:685–9. doi: 10.1002/ijc.2910280605, PMID: 7333703

[51] Darbre PD. Aluminium, antiperspirants and breast cancer. J Inorg Biochem. (2005) 99:1912–9. doi: 10.1016/j.jinorgbio.2005.06.001, PMID: 16045991

[52] Sultan SA Khan FA Wahab A Fatima B Khalid H Bahader A. Assessing leaching of potentially hazardous elements from cookware during cooking: A serious public health concern. Toxics. (2023) 11:1–14. doi: 10.3390/toxics11070640, PMID: 37505605 PMC10386729

[53] Exley C. Human exposure to aluminium. Environ Sci: Processes Impacts. (2013) 15:1807–16. doi: 10.1039/C3EM00374D, PMID: 23982047

[54] Choe E Min DB. Mechanisms and factors for edible oil oxidation. Compr Rev Food Sci Food Safety. (2006) 5:169–86. doi: 10.1111/j.1541-4337.2006.00009.x

[55] Giovannucci E Harlan DM Archer MC Bergenstal RM Gapstur SM Habel LA. Diabetes and cancer: a consensus report. CA: Cancer J Clin. (2010) 60:207–21. doi: 10.3322/caac.20078, PMID: 20554718

[56] Cohen S Janicki-Deverts D Doyle WJ Miller GE Frank E Rabin BS. Chronic stress, glucocorticoid receptor resistance, inflammation, and disease risk. Proc Natl Acad Sci. (2012) 109:5995–9. doi: 10.1073/pnas.1118355109, PMID: 22474371 PMC3341031

[57] Sephton SE Sapolsky RM Kraemer HC Spiegel D. Diurnal cortisol rhythm as a predictor of breast cancer survival. J Natl Cancer Inst. (2000) 92:994–1000. doi: 10.1093/jnci/92.12.994, PMID: 10861311

[58] Manouchehri E Taghipour A Ghavami V Ebadi A Homaei F Latifnejad Roudsari R. Night-shift work duration and breast cancer risk: an updated systematic review and meta-analysis. BMC Women’s Health. (2021) 21:1–6. doi: 10.1186/s12905-021-01233-4, PMID: 33653334 PMC7927396

[59] Wegrzyn LR Tamimi RM Rosner BA Brown SB Stevens RG Eliassen AH. Rotating night-shift work and the risk of breast cancer in the nurses’ health studies. Am J Epidemiol. (2017) 186:532–40. doi: 10.1093/aje/kwx140, PMID: 28541391 PMC5856106

[60] Cordina-Duverger E Menegaux F Popa A Rabstein S Harth V Pesch B. Night shift work and breast cancer: a pooled analysis of population-based case–control studies with complete work history. Eur J Epidemiol. (2018) 33:369–79. doi: 10.1007/s10654-018-0368-x, PMID: 29464445

[61] Stevens RG Brainard GC Blask DE Lockley SW Motta ME. Breast cancer and circadian disruption from electric lighting in the modern world. CA: Cancer J Clin. (2014) 64:207–18. doi: 10.3322/caac.21218, PMID: 24604162 PMC4038658

[62] Ward EM Germolec D Kogevinas M McCormick D Vermeulen R Anisimov VN. Carcinogenicity of night shift work. Lancet Oncol. (2019) 20:1058–9. doi: 10.1016/S1470-2045(19)30455-3, PMID: 31281097

[63] Islami F Goding Sauer A Miller KD Siegel RL Fedewa SA Jacobs EJ. Proportion and number of cancer cases and deaths attributable to potentially modifiable risk factors in the United States. CA: Cancer J Clin. (2018) 68:31–54. doi: 10.3322/caac.21440, PMID: 29160902

[64] Liu Y Nguyen N Colditz GA. Links between alcohol consumption and breast cancer: a look at the evidence. Women’s Health. (2015) 11:65–77. doi: 10.2217/WHE.14.62, PMID: 25581056 PMC4299758

[65] Rustagi AS Scott CG Winham SJ Brandt KR Norman AD Jensen MR. Association of daily alcohol intake, volumetric breast density, and breast cancer risk. JNCI Cancer Spectrum. (2021) 5:pkaa124. doi: 10.1093/jncics/pkaa124, PMID: 33733051 PMC7952225

[66] Gaudet MM Gapstur SM Sun J Diver WR Hannan LM Thun MJ. Active smoking and breast cancer risk: original cohort data and meta-analysis. J Natl Cancer Inst. (2013) 105:515–25. doi: 10.1093/jnci/djt023, PMID: 23449445

[67] Jones ME Schoemaker MJ Wright LB Ashworth A Swerdlow AJ. Smoking and risk of breast cancer in the Generations Study cohort. Breast Cancer Res. (2017) 19:1–4. doi: 10.1186/s13058-017-0908-4, PMID: 29162146 PMC5698948

[68] White AJ D’Aloisio AA Nichols HB DeRoo LA Sandler DP. Breast cancer and exposure to tobacco smoke during potential windows of susceptibility. Cancer Causes Control. (2017) 28:667–75. doi: 10.1007/s10552-017-0903-1, PMID: 28523418 PMC5530373

[69] Loomis D Guyton K Grosse Y El Ghissasi F Bouvard V Benbrahim-Tallaa L. Carcinogenicity of lindane, DDT, and 2, 4-dichlorophenoxyacetic acid. Lancet Oncol. (2015) 16:891–2. doi: 10.1016/S1470-2045(15)00081-9, PMID: 26111929

[70] Cohn BA La Merrill M Krigbaum NY Yeh G Park JS Zimmermann L. DDT exposure in *utero* and breast cancer. J Clin Endocrinol Metab. (2015) 100:2865–72. doi: 10.1210/jc.2015-1841, PMID: 26079774 PMC4524999

[71] Rodgers KM Udesky JO Rudel RA Brody JG. Environmental chemicals and breast cancer: An updated review of epidemiological literature informed by biological mechanisms. Environ Res. (2018) 160:152–82. doi: 10.1016/j.envres.2017.08.045, PMID: 28987728

[72] Dodson RE Nishioka M Standley LJ Perovich LJ Brody JG Rudel RA. Endocrine disruptors and asthma-associated chemicals in consumer products. Environ Health Perspectives. (2012) 120:935–43. doi: 10.1289/ehp.1104052, PMID: 22398195 PMC3404651

[73] Barr L Metaxas G Harbach CA Savoy LA Darbre PD. Measurement of paraben concentrations in human breast tissue at serial locations across the breast from axilla to sternum. J Appl Toxicol. (2012) 32:219–32. doi: 10.1002/jat.1786, PMID: 22237600

[74] Sivamani RK Jagdeo JR Elsner P Maibach HI eds. Cosmeceuticals and active cosmetics. Florida, U.S.A: CRC Press (2015). doi: 10.1201/b18895

[75] Swenberg JA Moeller BC Lu K Rager JE Fry RC Starr TB. Formaldehyde carcinogenicity research: 30 years and counting for mode of action, epidemiology, and cancer risk assessment. Toxicol Pathol. (2013) 41:181–9. doi: 10.1177/0192623312466459, PMID: 23160431 PMC3893912

[76] Collaborative Group on Hormonal Factors in Breast Cancer. Breast cancer and breastfeeding: collaborative reanalysis of individual data from 47 epidemiological studies in 30 countries, including 50–302 women with breast cancer and 96–973 women without the disease. Lancet. (2002) 360:187–95. doi: 10.1016/S0140-6736(02)09454-0, PMID: 12133652

[77] Barry JA Azizia MM Hardiman PJ. Risk of endometrial, ovarian and breast cancer in women with polycystic ovary syndrome: a systematic review and meta-analysis. Hum Reprod. (2014) 20:748–58. doi: 10.1093/humupd/dmu012, PMID: 24688118 PMC4326303

[78] Mørch LS Skovlund CW Hannaford PC Iversen L Fielding S Lidegaard Ø. Contemporary hormonal contraception and the risk of breast cancer. New Engl J Med. (2017) 377:2228–39. doi: 10.1056/NEJMoa1700732, PMID: 29211679

[79] Westhoff CL Pike MC. Hormonal contraception and breast cancer. Contraception. (2018) 98:171–3. doi: 10.1016/j.contraception.2018.05.002, PMID: 30193687 PMC6666389

[80] Bjornsdottir HH Rawshani A Rawshani A Franzén S Svensson A-M Sattar N. A national observation study of cancer incidence and mortality risks in type 2 diabetes compared to the background population over time. Sci Rep. (2020) 10:17376. doi: 10.1038/s41598-020-73668-y, PMID: 33060631 PMC7566479

[81] De Silva S Tennekoon KH Karunanayake EH. Overview of the genetic basis toward early detection of breast cancer. Breast Cancer: Targets Ther. (2019) 11:71–80. doi: 10.2147/BCTT.S185870, PMID: 30718964 PMC6345186

[82] Subaşıoğlu A Güç ZG Gür EÖ Tekindal MA Atahan MK. Genetic, surgical and oncological approach to breast cancer, with BRCA1, BRCA2, CDH1, PALB2, PTEN and TP53 variants. Eur J Breast Health. (2023) 19:55. doi: 10.4274/ejbh.galenos.2022.2022-7-2, PMID: 36605468 PMC9806937

[83] Abonyi-Tóth Z Rokszin G Fábián I Kiss Z Jermendy G Kempler P. Incident cancer risk in patients with incident type 2 diabetes mellitus in Hungary (Part 1). Cancers. (2024) 16:1745. doi: 10.3390/cancers16132414, PMID: 39001476 PMC11240453

[84] Abonyi-Tóth Z Rokszin G Sütő G Fábián I Kiss Z Jermendy G. Incident Cancer risk of patients with prevalent type 2 diabetes mellitus in Hungary (Part 2). Cancers. (2024) 16:2414. doi: 10.3390/cancers16132414, PMID: 39001476 PMC11240453

[85] Wang M Yang Y Liao Z. Diabetes and cancer: Epidemiological and biological links. World J Diabetes. (2020) 11:227. doi: 10.4239/wjd.v11.i6.227, PMID: 32547697 PMC7284016

[86] Shiovitz S Korde LA. Genetics of breast cancer: a topic in evolution. Ann Oncol. (2015) 26:1291–9. doi: 10.1093/annonc/mdv022, PMID: 25605744 PMC4478970

[87] Saeed S Asim M Sohail MM. Fears and barriers: problems in breast cancer diagnosis and treatment in Pakistan. BMC Women’s Health. (2021) 21:1–0. doi: 10.1186/s12905-021-01293-6, PMID: 33853583 PMC8045297

[88] Collaborative Group on Hormonal Factors in Breast Cancer. Menarche, menopause, and breast cancer risk: individual participant meta-analysis, including 118–964 women with breast cancer from 117 epidemiological studies. Lancet Oncol. (2012) 13:1141–51. doi: 10.1016/S1470-2045(12)70425-4, PMID: 23084519 PMC3488186

[89] King GL Park K Li Q. Selective insulin resistance and the development of cardiovascular diseases in diabetes: the 2015 Edwin Bierman Award Lecture. Diabetes. (2016) 65:1462–71. doi: 10.2337/db16-0152, PMID: 27222390 PMC4878431

[90] Lee H Ghebre R Le C Jang YJ Sharratt M Yee D. Mobile phone multilevel and multimedia messaging intervention for breast cancer screening: pilot randomized controlled trial. JMIR mHealth uHealth. (2017) 5:e7091. doi: 10.2196/mhealth.7091, PMID: 29113961 PMC5698632

[91] Goding Sauer A Siegel RL Jemal A Fedewa SA. Current prevalence of major cancer risk factors and screening test use in the United States: disparities by education and race/ethnicity. Cancer Epidemiol Biomarkers Prev. (2019) 28:629–42. doi: 10.1158/1055-9965.EPI-18-1169, PMID: 30944145

[92] Arem H Loftfield E. Cancer epidemiology: a survey of modifiable risk factors for prevention and survivorship. Am J Lifestyle Med. (2018) 12:200–10. doi: 10.1177/1559827617700600, PMID: 30202392 PMC6124966

